# Intraoperative Transesophageal Echocardiographic Guidance in Cardiac Surgery

**DOI:** 10.3390/jcdd12030093

**Published:** 2025-03-04

**Authors:** Yannis Dimitroglou, Antonios Karanasos, Andreas Katsaros, Argyro Kalompatsou, Grigorios Tsigkas, Konstantinos Toutouzas, Costantinos Tsioufis, Constantina Aggeli, Periklis Davlouros

**Affiliations:** 1 1st Department of Cardiology, University of Athens, Hippokration Hospital, 11527 Athens, Greece; dimiyann@med.uoa.gr (Y.D.); irokalomp@med.uoa.gr (A.K.); ktoutouz@med.uoa.gr (K.T.); ktsioufis@hippocratio.gr (C.T.); kaggeli@med.uoa.gr (C.A.); 2 School of Medicine, Patras University Hospital, 26504 Patras, Greece; gregtsig@upatras.gr (G.T.); pdav@upatras.gr (P.D.); 3 Department of Cardiac Surgery, Hippokration Hospital, 11527 Athens, Greece; ankatshome@hotmail.com

**Keywords:** transesophageal echocardiography, 3D echocardiography, cardiac surgery, intraoperative guidance

## Abstract

Transesophageal echocardiography (TEE) is a valuable tool for diagnosing structural heart diseases, offering superior resolution compared to transthoracic echocardiography. It allows for real-time evaluation of cardiac valves and both systolic and diastolic heart function. Additionally, TEE facilitates the prompt detection of potential complications during cardiac surgeries, such as paravalvular leaks, iatrogenic aortic dissections, and pericardial effusions. Advances in imaging, including 3D echocardiography, have further enhanced the visualization of complex structures like cardiac valves, providing “surgical views” that improve preoperative planning. These features have also made TEE indispensable for postoperative evaluation of cardiac valve repairs and for intraoperative guidance during minimally invasive procedures. This review article aims to summarize the indications for using TEE as an intraoperative tool in cardiac surgery.

## 1. Introduction

Transesophageal echocardiography (TEE) is an imaging modality used to assess cardiovascular structures with greater spatial and temporal resolution compared to transthoracic echocardiography [1]. Advantages of TEE include the proximity of the probe to the left atrium and the mitral apparatus, as well as the absence of structures that hinder ultrasound penetration. TEE also permits real-time acquisition of 2D and 3D images that can provide monitoring and guidance in the operation room [2]. Therefore, intraoperative TEE was already performed in the past and could modify surgical decisions aiming to minimize complications and improve outcomes [3,4].

Cardiac surgery has evolved from traditional open procedures to minimally invasive interventions that demand optimized outcomes. The dynamic nature of these procedures introduces additional challenges, requiring real-time guidance and decision making [5]. For these reasons, the indications for intraoperative TEE in cardiac operating rooms have expanded.

This review article aims to summarize the indications for using TEE as an intraoperative guide in cardiac surgery procedures.

## 2. General Principles of Intraoperative Transesophageal Echocardiographic Guidance in Cardiac Surgery

The application of TEE in the cardiac operating room requires close coordination between the anesthesiologist, the imaging expert—typically, a cardiologist—and the cardiac surgeon [6]. Communication must be swift and focused on the clinical scenario, making TEE training essential for the entire team to achieve optimal outcomes [7,8]. Additionally, discussions between the imaging and surgical teams are necessary to define treatment goals and plan the procedure effectively. In some cases, a preprocedural TEE may also be performed.

Probe insertion can be challenging and requires specialized training to minimize potential complications [9]. Once the probe is inserted, it is essential to acquire and confirm the necessary views before the procedure begins. Further discussion and confirmation of each procedural step are often needed. Intraoperative TEE provides valuable information on cardiac anatomy, patient volume status, and the systolic function of both the left and right ventricles. It also aids in the early detection of complications and can identify the presence of pericardial fluid. To facilitate effective communication, the TEE screen should be visible to every member of the surgical team, and brief communication codes and predefined protocols should be followed throughout the procedure and in the event of complications [10].

TEE is primarily used to guide cardiac valve repair procedures during surgery. However, it is also indicated in other cases, such as for patients with coronary artery disease, aortic disease, or those requiring mechanical circulatory support [5]. When compared to transthoracic echocardiography (TTE), TEE offers improved imaging primarily of the posterior anatomical structures, including the left atrium and the mitral valve. It is also superior in depicting anatomical structures and, owing to the improved stability of the probe in intubated patients, has improved 3D imaging characteristics [11,12]. In comparison, TTE is also significant in preprocedural and postprocedural evaluation of surgical patients. It offers natural and improved analysis of the intracardiac pressures that are altered in intubated patients owing to the increased intrathoracic pressure and anesthesia. In addition, evaluation of the aortic valve gradient, a relatively straightforward procedure with TTE, is often challenging with TEE, requiring expertise with deep transgastric views [13,14].

## 3. Mitral Valve Disease

**Figure 1:**
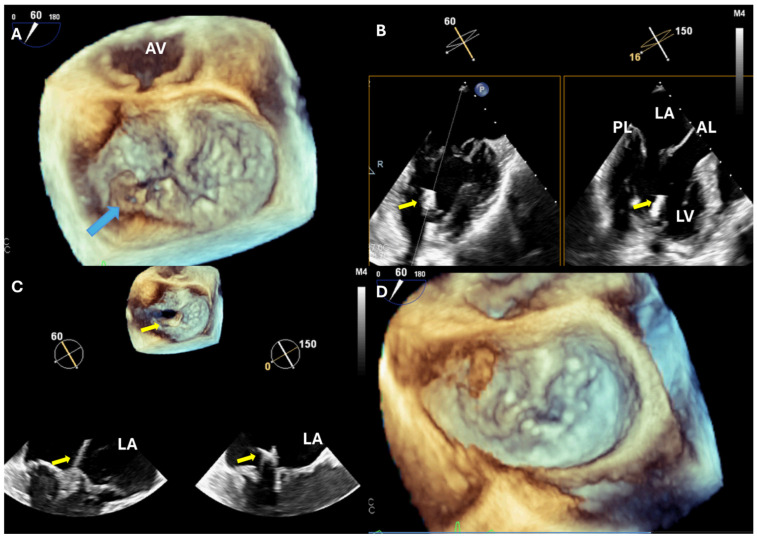
Beating-heart transapical Neochord implantation. (**A**) Preoperative assessment of the MV. Note the flail segment in the later part of the P2 (blue arrow). (**B**) The device (yellow arrow) is introduced into the left ventricle transapically and, using TEE, it is guided within the left atrium. (**C**) With the device in the left atrium, TEE provides guidance for the proper placement of the chords. (**D**) Postoperative assessment of the mitral valve. Note the absence of the residual flail part. AL: anterior mitral leaflet; AV: aortic valve; LA: left atrium; LV: left ventricle; MV: mitral valve; PL: posterior mitral leaflet.

The mitral valve (MV) is a complex, three-dimensional structure located in close proximity to the esophagus, making TEE—especially 3D-TEE—an indispensable tool for intraoperative guidance, particularly during mitral valve repair procedures [15]. According to the 2021 European Society of Cardiology (ESC) guidelines on the management of cardiac valve disease, mitral valve repair is the preferred approach for patients with primary mitral regurgitation (MR) who are not at high surgical risk [16]. Mitral valve replacement is reserved for cases where the valve’s anatomy is unsuitable for repair or when repair fails to sufficiently reduce MR. In addition, beating-heart transapical Neochord implantation under TEE guidance is a minimally invasive procedure that has demonstrated favorable medium-term outcomes in high-risk patients with primary MR, particularly those with flail segments—most notably in the P2 segment—and an increased coaptation index [17,18] (Figure 1).

**Figure 2:**
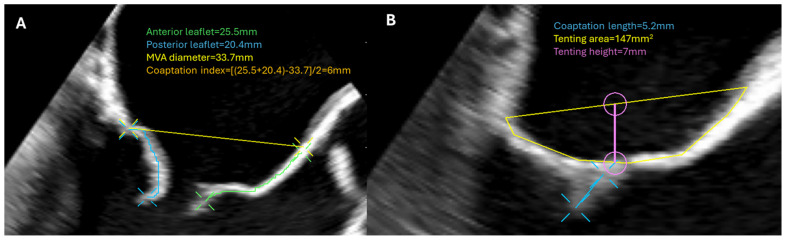
Essential echocardiographic measurements in patients with MR. (**A**) In the left panel is a patient with severe primary MR. Measurement of the leaflet length and annulus diameter as well as the calculation of the coaptation index can predict a sufficient coaptation length after an MV repair operation. These measurements are more important in operations without the availability of annular ring placement, as is the case in transapical chord implantation interventions. (**B**) In the right panel, there is a patient with moderate to severe secondary MR. Increased tenting area and tenting height are associated with increased recurrence rate and worse prognosis after isolated annuloplasty. MR: mitral regurgitation; MV: mitral valve; MVA: mitral valve area.

TEE plays a critical role in the analysis of mitral valve anatomy both preoperatively and postoperatively. The mid-esophageal bi-commissural view, combined with biplane imaging, allows for detailed assessment of the lateral, mid, and septal portions of the mitral apparatus to identify the mechanism of MR [19]. Key measurements include the leaflet length, annular dimensions, coaptation length, coaptation index, and tenting area (Figure 2). These parameters help determine the preferred treatment options and provide an estimate of the likelihood of achieving optimal anatomic outcomes. 3D echocardiography can enhance the capabilities of TEE, offering improved and more detailed analysis of the MV [20].

TEE is also the primary method for evaluating MR severity before and after surgical intervention by employing qualitative, semi-quantitative, and quantitative techniques. Color Doppler imaging is widely used, with vena contracta (VC) width and proximal isovelocity surface area (PISA) measurements serving as key indicators of MR severity [21]. However, these methods become less reliable in cases of multiple or eccentric jets. In such scenarios, 3D color Doppler TEE and 3D-PISA or 3D vena contracta measurements may provide improved accuracy, although their feasibility during time-sensitive intraoperative conditions is often limited [22]. When conventional methods are challenging, pulmonary vein flow patterns can offer a less precise but more reliable estimation of the MR grade [23]. Additionally, careful consideration of preload and afterload conditions is essential, as elevated blood pressure can lead to overestimation of MR severity on color Doppler. Close coordination with the anesthesiologist is critical to ensure accurate assessments under stable hemodynamic conditions. Measurement of the velocity time integral (VTI) of transmitral flow provides an estimate of regurgitant volume. This approach is particularly useful in patients without concurrent aortic regurgitation (AR) and allows for the calculation of the regurgitant fraction to evaluate MV severity [24]. However, the accuracy of this method diminishes when the mitral annulus area is altered during surgery, as the calculations are highly dependent on the annular dimensions.

**Figure 3:**
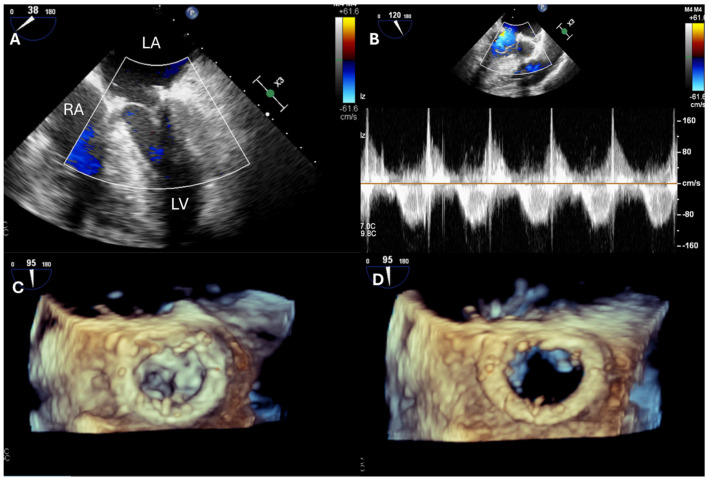
Postoperative assessment of a patient undergoing mitral valve repair with chord implantation and ring placement for primary MR. (**A**) Using color Doppler, TEE can assess for residual MR or the presence of paravalvular leaks, which were excluded in this case. (**B**) With continuous Doppler, the transmitral flow is recorded, and the mean gradient can be measured. (**C**,**D**) Using a 3D view, TEE can visualize the mitral valve from the “surgical views” with the aorta in the 12th h and the left atrial appendage in the 9th h. In this case, no residual flail segment was noted, and the opening of the mitral valve was also anatomically sufficient. LA: left atrium; LV: left ventricle; MR: mitral regurgitation; RA: right atrium.

Postoperative assessment for iatrogenic mitral stenosis (MS) is crucial. The mean gradient across the valve is the simplest method for evaluation, with values above 5–6 mmHg suggesting stenotic physiology [25,26,27]. However, these measurements can be influenced by cardiac output and heart rate, making them less reliable in certain cases. In more complex scenarios, 3D-TEE can provide a direct measurement of the mitral valve area, which holds prognostic significance for clinical outcomes [28] (Figure 3).

In patients undergoing mitral valve replacement, intraoperative TEE remains valuable for assessing leaflet motion and functionality. Using a 3D surgical view combined with color Doppler, TEE can identify or exclude significant paravalvular leaks, ensuring optimal surgical results and guiding any necessary corrections [29].

## 4. Tricuspid Valve Disease

The tricuspid valve (TV), once referred to as the “forgotten valve”, is now frequently addressed, particularly in patients undergoing left-sided surgeries or, less commonly, for severe primary tricuspid regurgitation (TR). While TR is most often secondary to volume overload, primary TR or mixed cases can also be identified using TEE. Although the TV is typically tricuspid, anatomical variants with four or even five leaflets are not uncommon, with double septal or posterior leaflets being the ones most frequently observed [30,31].

Preoperative and intraoperative TEE provides detailed visualization of TV anatomy and its relationship to adjacent structures, which serve as anatomic landmarks for leaflet identification. The septal leaflet is adjacent to the interventricular septum, and the anteroseptal commissure lies near the non-coronary cusp of the aortic valve (AV). Additionally, the anterior papillary muscle acts as the landmark for the anteroposterior commissure, while the coronary sinus corresponds to the posteroseptal commissure [32]. From mid-esophageal views between 50 and 90°, the anterior and posterior leaflets are typically visible. By employing biplane imaging, coaptation views of the septal leaflet with either the anterior or the posterior leaflet can also be obtained [33]. Transgastric views offer an en-face perspective of the tricuspid valve, which can alternatively be achieved using 3D-TEE from distal–esophageal views [34]. It is worth noting that the leaflets are visualized better from esophageal views during systole and from transgastric views during diastole. This comprehensive imaging assessment is instrumental in accurately defining TV anatomy and guiding surgical interventions.

Important measurements for determining the need for TV intervention in patients undergoing left-sided surgery include the assessment of the tricuspid annulus and the severity of TR. According to current guidelines, a tricuspid annulus end-diastolic diameter greater than 40 mm, measured in a four-chamber view, is considered dilated, and, in these patients, intervention is indicated [5]. 3D-TEE enhances this evaluation by allowing for measurements in additional planes through multiplanar reconstruction. Intervention is also indicated in patients with severe TR undergoing left sided surgery [35]. In such cases, TV repair with ring placement is the preferred approach. The evaluation of TR severity follows similar principles to those used for MR [23]. However, TR severity is significantly affected by right ventricular (RV) preload and afterload [36]. Therefore, assessments should be performed both preoperatively and postoperatively under conditions of stable pulmonary artery pressure and volume status. Additionally, studies have demonstrated a significant association between postoperative TV stenosis and adverse outcomes [37]. Consequently, Doppler studies and 3D analysis are essential in the postoperative period to exclude the presence of TV stenosis and ensure favorable clinical results. Lately, the range of TV repair procedures has been expanding, with increasing reports of minimally invasive procedures in which TEE guidance is essential. Such procedures include annuloplasty using pericardial patches, edge-to-edge artificial chordae placement, papillary muscle plasty, and bicuspidization and cleft closure, and they have demonstrated favorable results [38,39].

## 5. Aortic Valve Disease

The aortic valve is typically tricuspid, and calcific aortic stenosis (AS) represents the most common cause of clinically significant valvular heart disease, particularly in the aging population [40]. For severe AS, transcatheter aortic valve implantation (TAVI) is the preferred treatment for patients over 75 years of age or those at increased surgical risk. Surgical intervention for AS is generally reserved for patients under 75 years of age with low surgical risk or cases involving unfavorable aortic valve anatomy or concomitant coronary artery disease (CAD) requiring a surgical approach [16].

**Figure 4:**
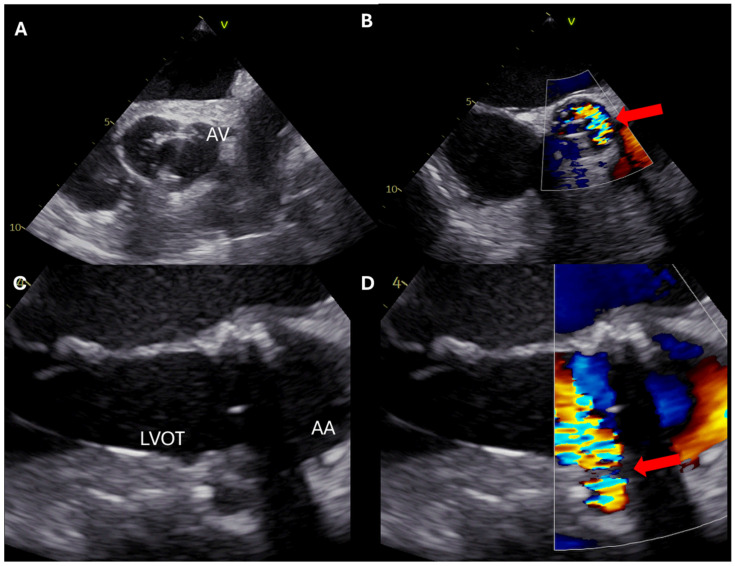
Postoperative assessment of a patient undergoing bioprosthetic aortic valve implantation. (**A**,**B**) Short-axis views. (**C**,**D**) Long-axis views. A significant paravalvular leak (red arrow) is noted between the 1st and the 4th h in the anatomical position of the left aortic cusp. Based on the TEE findings, a new valve was implanted. AA: ascending aorta; AV: aortic valve; LVOT: left ventricular outflow tract.

**Figure 5:**
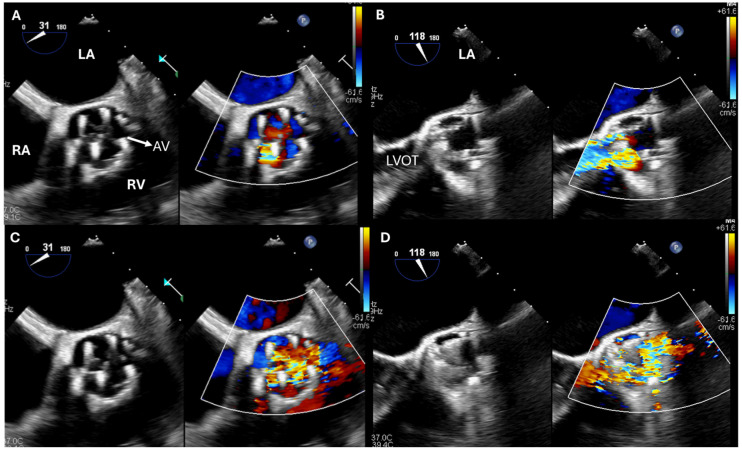
Postoperative assessment of a patient undergoing bioprosthetic aortic valve replacement. (**A**) Diastolic frames showing paravalvular leak in the 7th h. (**B**) Diastolic frame from long-axis views showing significant regurgitation. (**C**,**D**) Systolic frames showing aliasing, which indicated stenotic function of the valve. Based on the TEE findings, a new valve was implanted. AV: aortic valve; LA: left atrium; LVOT: left ventricular outflow tract; RA: right atrium; RV: right ventricle.

In patients undergoing aortic valve replacement for AS, preoperative TEE is crucial for measuring aortic annulus dimensions to select the appropriate valve size [41]. Intraoperatively, TEE plays a pivotal role in assessing the correct placement of the prosthetic aortic valve and identifying potential paravalvular leaks [42] (Figure 4 and Figure 5). Additionally, measurement of the maximum aortic valve velocity and the transvalvular pressure gradient can be performed using deep transgastric views when feasible [3,43]. Significantly increased velocities may suggest prosthetic valve stenosis or subvalvular obstruction, aiding in prompt diagnosis and intervention.

In patients with AR, no universally accepted echocardiographic index exists to precisely define the severity of valve disease [44]. Therefore, even in patients with multiple valve disease or concomitant CAD or disease of the aorta, the evaluation of AR severity and confirmation of indication for intervention should ideally be performed prior to surgery, including cases requiring emergent intervention. However, intraoperative TEE plays a significant role in identifying the mechanisms of AR and guiding aortic valve repair in suitable cases, such as patients with a prolapsed aortic cusp [45]. Using the biplane mode from short-axis views, the coaptation of the three cusps can be assessed individually. Alternatively, 3D-TEE with multiplanar reconstruction provides a more detailed and accurate analysis of cusp anatomy and function [46]. Postoperatively, TEE is essential to confirm the success of aortic valve repair by ensuring minimal or no residual AR and the absence of persistent cusp prolapse [47]. Additionally, the coaptation length between the cusps should measure at least 4 mm to improve the likelihood of a durable surgical outcome [48].

## 6. Coronary Artery Disease

In patients with CAD undergoing coronary artery bypass grafting (CABG) surgery, the role of intraoperative TEE differs between those with chronic CAD and those presenting with acute coronary syndromes (ACS). In chronic CAD, left ventricular (LV) function and the presence of valvular heart disease are typically assessed in advance of the surgery and the heart team meeting. Conversely, in patients with ACS, preoperative TEE following a stepwise approach can significantly improve surgical outcomes. However, even in patients undergoing isolated planed CABG, intraoperative TEE has been shown to reduce the risk of unplanned valve intervention and improve mortality rates [49].

**Figure 6:**
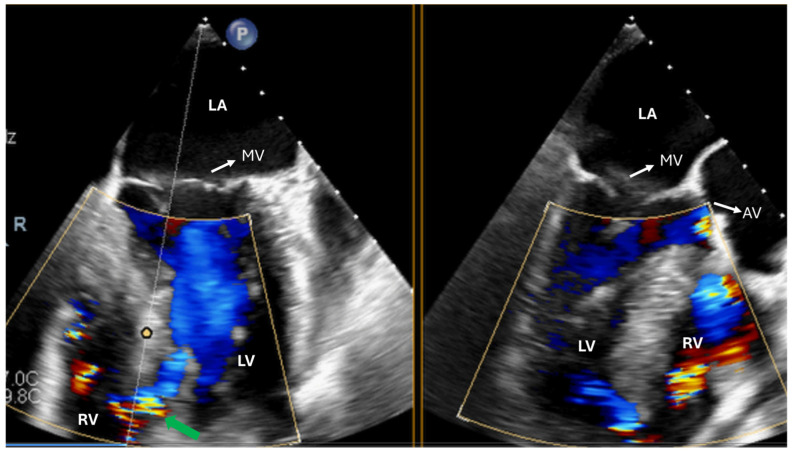
Assessment of a patient undergoing CABG for anterior STEMI with late presentation and 3-vessel disease on cardiac catheterization. Preoperative assessment revealed a ventricular septal defect (VSD) at the apical part of the intraventricular septum (green arrow). Subsequently, a septal patch was placed, and postoperative assessment revealed complete closure of the VSD. AV: aortic valve; LA: left atrium; LV: left ventricle; MV: mitral valve; RV: right ventricle.

Preoperatively, TEE provides critical information about LV function, including the identification of aneurysmal LV segments, which can guide surgical decision making [50]. Additionally, ACS-related complications, such as interventricular septal defects, can be detected and addressed prior to surgery [51] (Figure 6). Careful evaluation of cardiac valve function is essential, as the presence of moderate to severe AS or MR may necessitate concurrent aortic valve replacement or mitral valve repair/replacement during CABG [49].

Postoperatively, TEE is used to compare LV function with preoperative findings to identify any new areas of LV hypokinesis [50,52]. Moreover, an assessment of both LV and RV function is vital, as significant dysfunction may indicate the need for mechanical circulatory support. In addition, newer cardiovascular modalities, including semi-automatic speckle tracking echocardiography or myocardial work, have been utilized for the evaluation of ventricular function, with promising results [53,54,55]. Postoperative evaluation of valvular function is also critical.

## 7. Diseases of the Aorta

Intraoperative TEE is a critical tool for patients undergoing interventions for aortic aneurysms, particularly those involving the aortic root and the ascending aorta, as well as for those with aortic dissections [4]. As in other surgeries involving cardiopulmonary bypass, TEE plays a vital role in identifying the optimal site for aortic cannulation, excluding areas with extensive atherosclerosis [56]. In aortic root surgeries, special care is required to preserve the coronary arteries. Using color Doppler with TEE from both short-axis and long-axis views of the aortic root helps identify iatrogenic coronary artery occlusion [57]. TEE can also be used for preoperative and postoperative assessment and guidance in patients undergoing minimally invasive aortic root surgeries with aortic valve sparing (David procedure) [58].

In patients with aortic dissections, intraoperative TEE is invaluable for assessing the extent of the dissection, accurately localizing the entry tear, and detecting complications, such as AR and pericardial effusion [59]. When AR is present, TEE can define the underlying mechanism and guide the appropriate treatment approach. Additionally, TEE can identify rare but life-threatening complications, such as iatrogenic dissection or endoleaks of aortic grafts, ensuring prompt intervention when necessary [60].

## 8. Other Indications

### 8.1. Pulmonary Valve Disease and Adult Congenital Heart Disease

Pulmonary valve disease, including pulmonary stenosis and regurgitation, is most commonly congenital or a long-term complication of surgical correction for congenital heart disease [61]. In patients undergoing pulmonary valve replacement, intraoperative TEE is essential for excluding post-surgical complications, such as pulmonary stenosis or residual pulmonary regurgitation. Furthermore, in patients with congenital aortic stenosis undergoing the Ross procedure, intraoperative TEE plays a critical role in assessing pulmonary autograft performance by excluding neoaortic regurgitation and ensuring the preservation of coronary arteries, thereby contributing to the success of the procedure [62,63].

### 8.2. Hypertrophic Cardiomyopathy

In patients with hypertrophic obstructive cardiomyopathy, surgical septal myectomy may be considered for symptomatic individuals with significant left ventricular outflow tract (LVOT) obstruction despite optimal medical therapy, including beta-blockers, calcium channel blockers, and the cardiac myosin inhibitor mavacamten [64]. In addition to its role in interventional procedures, in such cases requiring surgery, intraoperative TEE is significant in delineating the anatomy of the interventricular septum and assessing the systolic anterior motion (SAM) of the mitral valve, facilitating precise planning and execution of the surgical procedure [65,66]. In addition, 3D TEE may facilitate understanding of MV anatomy in patients with SAM and significant MR [67]. Postoperatively, TEE is crucial for evaluating the adequacy of the myectomy by measuring the LVOT gradient and identifying potential complications, such as iatrogenic ventricular septal defects (VSD) [68]. The LVOT gradients may require pharmaceutical provocative testing with isoproterenol or dobutamine, as intraoperatively measured gradients tend to be underestimated with TEE [69]. The presence of a VSD should be assessed using mid-esophageal long-axis views or five-chamber views and transgastric short-axis views.

### 8.3. Infective Endocarditis

In patients with infective endocarditis (IE), a comprehensive TEE study is performed before surgery to assess the extent of valvular dysfunction, estimate the size of the vegetations, and exclude the presence of IE complications including paravalvular abscesses [70]. Given that the progression of the infection may be rapid, intraoperative TEE provides anatomical reassessment to potentially improve outcomes [71]. Postoperatively, TEE is used to detect areas not adequately treated, paravalvular leaks, or significant valve regurgitation after vegetectomy, requiring valve replacement [72].

### 8.4. Mechanical Circulatory Support

Intraoperative TEE during the placement of mechanical circulatory assist devices plays a critical role in assessing cannulation sites, excluding the presence of thrombi, and ensuring proper device positioning. Additionally, it is essential to evaluate for significant AR and RV dysfunction, as both can worsen following the placement of left ventricular assist devices (LVADs) and lead to adverse outcomes [73]. Postoperatively, TEE is equally important for detecting intracardiac air, which must be excluded to prevent the risk of air embolism and associated complications [74].

### 8.5. Heart Transplantation

In patients undergoing orthotopic heart transplantation, TEE should assess LV and RV function pre- and postoperatively [75]. Preoperatively, it is important to exclude the presence of enlarged apical thrombi. Postoperatively, it is crucial to exclude the presence of LV or RV graft dysfunction [76]. Intraoperative, TEE may also be used to determine the suitability and good functioning of the anastomotic sites, including the great vessels and the left atrium. Postoperative evaluation of the function of cardiac valves is important because significant TR is not uncommon [5].

## 9. Conclusions

Intraoperative TEE has become an indispensable tool in cardiac surgeries, allowing for the assessment of surgical outcomes and the prompt recognition of complications (Table 1). With the growing focus on minimally invasive, beating-heart procedures aimed at repairing underlying cardiac pathology, TEE plays an increasingly critical role in surgical planning and intraoperative guidance. For these reasons, it is essential for cardiologists, anesthesiologists, and cardiac surgeons to be familiar with the procedures and the most commonly used transesophageal echocardiographic views. This shared expertise leads to improved collaboration and coordination in the operating room, ultimately resulting in better surgical and clinical outcomes.

## Figures and Tables

**Table 1:** Summary table of the preoperative and postoperative role of TEE in patients undergoing cardiac surgery.

	Preoperative	Postoperative
MV repair	Describe MR mechanism Measure leaflet length Measure annulus size Estimate MR severity	Ensure sufficient MR reduction No flail part Exclude iatrogenic MV stenosis
TV repair	Describe TR mechanism Describe TV anatomy Measure TV annulus	Exclude iatrogenic TV stenosis
AV repair	Describe mechanism of AR Define feasibility of repair Image coronary vessels Image ascending aorta	Ensure no/mild residual AR Exclude persistent prolapse Estimate durability of repair (measure coaptation length)
Prosthetic valves	Exclude concomitant pathologies (e.g., significant TR)	Exclude PVLs Exclude insufficient valve expansion
CABG	Exclude concomitant valvular heart disease Exclude MI complications	Estimate LV and RV function
Aortic root surgery	Guide aortic cannulation Define feasibility of valve sparing surgeries	Image coronary vessels Exclude iatrogenic dissection Exclude aortic graft endoleaks
Pulmonary valve	Estimate gradient and RV function	Exclude residual stenosis or regurgitation
Hypertrophic cardiomyopathy	Assess MV function and the extent of anterior leaflet SAM	Estimate postoperative gradient Exclude iatrogenic VSD
Infective endocarditis	Exclude complications (e.g., abscess) Reassess valvular function	Exclude residual valve regurgitation Assess prosthetic valve function
Mechanical circulatory support	Guide cannulation sites Exclude thrombi Guide positioning of the device	Estimate LV and RV function Exclude presence of air in the device
Heart transplantation	Determine suitability of anastomotic sites Exclude presence of thrombi	Estimate LV and RV function Exclude presence of significant TR

AV: aortic valve; CABG: coronary artery bypass grafting; LV: left ventricular; MI: myocardial infarction; MR: mitral regurgitation; MV: mitral valve; PVL: paravalvular leak; RV: right ventricular; TR: tricuspid regurgitation; TV: tricuspid valve; VSD: ventricular septal defect.

## Data Availability

Not applicable.
